# Immunomodulatory Arming Factors—The Current Paradigm for Oncolytic Vectors Relies on Immune Stimulating Molecules

**DOI:** 10.3390/ijms22169051

**Published:** 2021-08-22

**Authors:** Cole W. D. Peters, Fares Nigim

**Affiliations:** 1 Pediatric Hematology-Oncology, Los Angeles School of Medicine, University of California, Los Angeles, CA 90024, USA; colewpeters@yahoo.com; 2 Translational Neuro-Oncology Laboratory, Department of Neurology, Divisions of Neuro-Oncology and Hematology/Oncology, Massachusetts General Hospital, Harvard Medical School, Boston, MA 02114, USA; 3 Dana Farber Cancer Institute, Harvard Medical School, Boston, MA 02215, USA

**Keywords:** oncolytic viral vector, arming transgenes, cancer therapy, oncolytic virology

## Abstract

The dogma of engineering oncolytic viral vectors has shifted from emphasizing the viral lysis of individual cancer cells to the recruitment and coordination of the adaptive immune system to clear the tumor. To accomplish this, researchers have been adding several classes of transgenes to their preferred viral platforms. The most prevalent of these include antibodies and targeting moieties, interleukins and cytokines, and genes which rely on small molecule co-administration for tumor killing. Most current vectors rely exclusively on one of these types of transgenes to elicit the desired immune response to clear tumors, but are not mutually exclusive, with several larger OVs armed with several of these factors. The common theme of emerging armed vectors is to simply initiate or enhance infiltration of effector CD8+ T cells to clear the tumor locally at OV infection sites, and systemically throughout the body where the OV has not infected tumor cells. The precision of oncolytic vectors to target a cell type or tissue remains its key advantage over small-molecule drugs. Unlike chemo- and other drug therapies, viral vectors can be made to specifically infect and grow within tumor cells. This ensures localized expression of the therapeutic transgene to the diseased tissue, thereby limiting systemic toxicity. This review will examine the immunomodulating transgenes of current OVs, describe their general effect on the immune system, and provide the rationale for each vector’s use in clearing its targeted tumor.

## 1. Oncolytic Viruses

The key tenet for oncolytic vectors is to kill tumor cells while leaving non-tumor cells alive. This ideal has become less clear cut in recent years due to the discovery of tumor-associated macrophages and neovascular involvement in tumor growth [1,2], which can be targeted by OVs. However, a replicating vector’s primary directive must be to exploit the differences between cancerous and normal cell growth in order to replicate in cancer cells while remaining safe for patients [3]. To accomplish this, most vectors have deletions or mutations within their genomes which attenuate the virus in normal tissue. Recently, these empty spaces in attenuated vectors have become sites to ‘arm’ vectors with potent immunostimulatory transgenes. These ‘armed’ vectors express transgenes which increase tumor destruction and survival benefit over the parental vectors. Below, we briefly describe the main genes that are altered in the oncolytic viral vectors which will be covered to provide a better understanding of vector attenuation.

Oncolytic herpes simplex-1 (oHSV) vectors commonly remove the y34.5 genes to ablate the neurotoxicity of wildtype HSV1. These genes recruit protein phosphatase 1α to maintain eukaryotic initiation factor-2α in a de-phosphorylated state, which allows the translation of viral proteins during cell stress [4,5]. Many y34.5-deleted vectors contain an additional deletion of the ICP47/U_S_12 gene, which complements the y34.5 deletion through eliciting expression of the U_S_11 gene, while remaining attenuated in vivo (reviewed in [6]). Other oHSV vectors retain the y34.5 gene under a strict tissue-specific promoter, utilize miRNAs to control the expression of essential immediate early genes which prevent the lytic cycle of the virus in neural tissues, and/or re-engineer HSV1 entry proteins to limit infection to cells overexpressing tumor-associated proteins [7,8,9,10,11].

Oncolytic adenoviruses (oAds) have their E1A and E1B genes disabled, which prevent interaction with Retinoblastoma and p53 proteins, respectively. Deletions within E1A prevent Ad vectors from stimulating the S-phase of the cell cycle, limiting productive infection to rapidly growing tissues, namely, tumors [12]. Synergistically, E1B-55k suppresses p53 activity; thus mutations, in it further reduce permissive cells to those with p53 mutation or suppression [13]. Other common mutations include deletions within protein phosphatase binding E4orf4, ubiquitin ligase E4orf6, and the immune suppression genes of E3, which all inhibit viral replication in normal tissues. Another facet of modern Ad vectors is their use of a chimeric polymerase (E2b) gene and cell attachment fiber and penton regions, which restrict their infection to tumor tissues [12,14,15]. 

Vaccinia vectors (VACVs) are larger than herpes and adenovirus, and often have their thymidine kinase (TK) selectively mutated for attenuation. Current VACVs are based on vaccine strains for smallpox, such as the Wyeth strain, building off the extensive safety data available after worldwide vaccine drives, such as JX-594/Pexa-Vec [16]. Other VACVs are also in development, using Wyeth and other vaccine strains as their initial backbone. Incredibly, most of the vaccinia virus genome’s products are still yet to be studied in depth to ascertain their function. However, similar to HSV vectors with hinge region deletions, there are VACVs with large core regions of their genome completely removed, allowing multiple arming genes to be inserted (reviewed in [17,18]). 

Oncolytic RNA viruses are much smaller than their DNA counterparts. This ultimately means that they have less transgene capacity, which has only become a recent issue due to the limited enthusiasm of past researchers to add more than one arming transgene to their vector platform. The best characterized RNA vectors currently in study are Maraba, vesicular stomatitis virus (VSV), measles, Newcastle disease virus, polio, and reovirus. Maraba and VSV are both negative (−) ssRNA rhabdoviruses. However, methods to attenuate these vectors differ, with current Maraba vectors incorporating point mutations in the M and G genes, whereas VSV vectors either incorporate an interferon (IFN) sensitivity mutation M51delta or IFNβ itself as a transgene [19,20,21]. Newcastle disease virus (NDV) and measles (MV) vectors are both (−) ssRNA paramyxoviruses. NDV is an avian virus which is safe in humans, whereas measles vectors, such as VACV, are derived from a vaccine strain (Edmonston), discussed and reviewed in [22,23,24]. Similarly, the oncolytic positive(+) ssRNA polio vector currently in clinical trial, PVSRIPO, uses the Sabin vaccine strain with an insertion of a human rhinovirus sequence within the IRES [25]. However, PVSRIPO harbors no transgenes and will not be covered here, nor will other vectors that do not utilize arming factors that fall within our categories. Reolysin, a type-3 Dearing strain reovirus/rotavirus, is the only human virus found in the wild, fully replicative, and attenuated enough for use in patients. Unlike other RNA vectors, REOLYSIN has a dsRNA genome. Due to the vector’s sensitivity to Ras activity, it is being investigated in many cancers, and modified to compete with the emerging multi-armed vectors [26,27].

Although modern vectors are still selected for their ability to grow in tumor tissues and cell lines, the vector’s primary focus during engineering has shifted from quickly lysing tumor cells to the recruitment of anti-tumor immune infiltrates. Currently, the most sought after and common focus of arming genes are immune-modulatory molecules, or proteins which increase immune cell activity. This review will list many of the current vectors that utilize such transgenes, how their transgenes affect the immune system, and the status of their clinical trials.

## 2. Viral Vectors Armed with Antibodies and Other Targeting Moieties

No review covering oncolytic viruses can omit the impact that T-cell checkpoint inhibitors have had on the field. Their discovery has fundamentally shifted oncolytic virology towards engineering vectors in an immuno-oncology context. The “big three” inhibitors are antibodies that target program death ligand-1 (PDL1), its receptor PD1, and CD28 inhibitory ligand cytotoxic T-lymphocyte-associated protein 4 (CTLA4). All three antibodies bind their targets, preventing their inhibitory signals from ever reaching the T cell, thus maintaining an active, tumor-killing, effector cell [28]. These moieties can significantly improve patient outcomes, and similarly enhance OV efficacy in tumor models; thus, they are being utilized in tandem with OVs in multiple clinical trials (Table 1-Interventions). Local expression of checkpoint inhibitors through a vector offers a hope to reduce toxicity observed by the systemic delivery of inhibitor antibodies [29]. One pitfall stalling this improved safety hypothesis is the difficulty of demonstrating toxicity in mouse models, which can be systemically injected with anti-CTLA4 and anti-PD1 antibodies without observing the adverse events that occur with patients. Many current research efforts are looking into co-administration of checkpoint inhibitors with OV; however, this article will explore those which are engineered to express them. 

The first OV accepted for clinical use in the United States was the oHSV talimogene laherparepvec (TVEC), now IMLYGIC, for melanoma treatment after its success in the OPTiM study [30]. Moving to test its efficacy in combination with anti-CTLA4 or anti-PD1, multiple trials are currently ongoing. Early results suggested either agent in combination was well tolerated in patients [31,32]; however, recent cancellation of Masterkey-265 by Amgen, in February 2021, signaled that pembrolizumab (anti-PD1 antibody) by itself worked better than in combination with TVEC for melanoma [33]. Although unfortunate for TVEC aficionados, the ability of a vector to express its own checkpoint inhibitor offers the ability to restrict inhibitor antibody to areas immediately surrounding the tumor, enabling the recruitment and activation of immune cells immediate to the tumor. To accomplish this, several vectors which harbor checkpoint antibodies are being researched. Replimune, founded by the inventor of TVEC, has developed a new line of HSV1 “RP” vectors using the RH018A strain. These vectors express GM-CSF, have γ34.5 deletions, and immediate-early expression of U_S_11 similar to TVEC, but incorporate a fusogenic glycoprotein derived from gibbon ape leukemia virus (GALV-GP-R) to cause fusogenic cell death, which enhances tumor killing [34]. In addition, these RP vectors were armed with an anti-CTLA4 secretable antibody which further enhanced their tumor killing efficacy in mouse xenograft A20 lymphoma models. In theory, production of the antibody occurs at the tumor site, making its first targets infiltrating T cells. This additive boost is vital, because the tumor microenvironment actively inhibits T-cell killing through the tumor expression of PDL1. Tumor resident T_REG_ cells also secrete tolerizing agents such as IL-10 to inhibit the proliferation and activation of effector T cells [35]. Checkpoint inhibitor antibodies counteract some of this inhibition by restoring T cell effector function and some proliferation, even after T-cell exhaustion [36]. Interestingly, the CTLA4 therapeutic antibodies are IgG_1_ subtypes, whereas anti-PD1 and anti-PDL1 moieties are IgG_4_. Whether responses to either subtype affect tumor regression have not been thoroughly studied, but IgG_4_ stability and weaker interaction with FcRγ could play a noticeable role when expressed in the context of viral infection [37].

The ONCR177 vector, from Oncorus, is another herpes vector expressing checkpoint inhibitors. Unlike TVEC, ONCR177’s tumor targeting is transcriptionally based, relying on miRNA124 and miRNA177 targeting sites within the ICP4 and ICP8 essential HSV1 genes. In order to insert transgenes, the entire joint region, also known as the internal long and short repeats, was deleted within the vector. This leaves the remaining γ34.5 gene intact. Additionally, there are five alanine substitutions within the U_L_37 gene to abrogate retrograde transport through neurons [38]. The large deletion of the internal repeats opens up around 9 kb for the insertion of arming genes, which Oncorus has accomplished, adding both anti-CTLA4 and anti-PD1 antibodies as well as IL-12, FLT3LG, and CCL4 transgenes. In mouse tumor models, intra-tumoral injection resulted in complete responses, whereas no effect was observed when CD8+ T-cells or NK cells were depleted [39]. These results were used to begin clinical trials with the addition of anti-CTLA4 antibody. 

Anti-PD1/PDL1 treatments have less toxicity observed with them than CTLA4 therapy, which may explain why fewer researchers are looking to incorporate them into vectors. Two Newcastle disease virus (NDV) vectors expressing anti-PD1, anti-PDL1, or a fusion of them to the IL-12 cytokine (immunocytokine), are currently being tested. NDVs armed with these immunocytokines increased survival in both highly aggressive unilateral and bilateral B16-F10 murine melanoma models, with the immunocytokine offering slightly better benefits [40]. A myxoma virus vector, vPD1, also expresses the anti-PD1 antibody, which results in the clearance of B16-F10 melanomas in mice, dependent on CD8+ T cells, although interestingly performing better with CD4+ T-cell depletion [41]. Although difficult to study, the question of whether OVs can re-sensitize tumors to checkpoint blockade will be important to solve. Checkpoint blockades often lose their efficacy in many tumors over time; therefore, the ability of OV insult to regenerate T-cell responsiveness would be a much-welcomed effect. One clinical study is being performed on checkpoint blockade non-responders using armed OVs, which will hopefully yield good results (NCT03003676).

The expression of these T-cell-activating antibodies from the virus confines the antibody to zones of viral replication, which hypothetically will mitigate the adverse events observed in patients from systemic intravenous administration. This is a very important safety consideration for bi-specific T-cell engagers (BiTEs), which are quickly being integrated with OVs. BiTEs utilize the well-established technique of fusing antibody variable chains together in order to target two separate moieties, such as CD4 and CCR5, to inhibit HIV [42]. BiTEs differ in their design to activate T cells by targeting CD3 with an antagonizing antibody, while simultaneously targeting a tumor antigen. This brings an activated T cell to the tumor cell, soliciting anti-tumor activity (reviewed extensively in [43]). Expressing BiTEs at tumor sites also enables T cells with TCRs not specific for tumor antigen to nonetheless become active and target tumor tissues, regardless of the expression of MHC class I [44]. The local expression of the BiTE also mitigates problems associated with systemic administration, which have caused several BiTE trials to be put on hold due to adverse events [45]. The PsiOxus company have adopted BiTEs into their adenovirus platform. The base vector PsiOxis uses is the ColoAd1/Enadenotucirev (EnAd) oncolytic adenovirus, characterized by an Ad11p/3 chimeric E2B region, deletions in the E3 region, and a 24 bp deletion in E4orf4. EnAd-EPCAM BiTE expresses a CD3/EPCAM BiTE which activates human T cells to kill tumor cells in vitro and from ex vivo patient ascites [46]. PsiOxus have engineered several more vectors based off the EnAd-BiTE which express BiTEs and cytokine transgenes between the L5 and E4 genes. The BiTE itself is termed FAP-TAc, and targets tumor-associated fibroblasts by causing T cells to engage cells expressing the fibroblast-activating protein, once more highlighting that OVs can also be used to target non-transformed cancer-associated cells as well. NG641 is currently being used in two clinical trials (NCT04830592 and NCT04053283). Several other BiTE-OVs include CAdDuo-IL12-CD44v6 BiTE [47], REOLYSIN-124 expressing a CD3xTRP1 (tyrosinase-related protein-1) and CD3xHER2 [48], and a herpes vector expressing PDL1 and BiTE within a G207 backbone [49].

The local expression of immune-stimulatory antibodies from the above vectors were all derived from a common and central issue of today’s immunotherapy dogma. The infiltrating T cells that initially reduce tumor burden eventually become inert, stop proliferating, and stop killing tumor cells. Antibodies which prevent this inhibition are only one method of re-initiating effector T cell activity. Much like the next class of arming genes, the ingenuity and benefit of encoding an antibody or targeting moiety depends on secretion of the transgene to reach the immune cells upon which it depends. This means that the efficacy of the arming gene is dependent upon the infected cell to efficiently secrete the active form of these antibodies. 

## 3. Vectors Expressing Cytokines and Immune-Activating Molecules

The next approach being actively tested for armed OVs is to engineer the expression of interleukins and cytokines that activate and cause effector differentiation. Expression of granulocyte macrophage colony-stimulating factor (GM-CSF) is the most notable example of this. GM-CSF attracts monocytes and causes their differentiation into antigen-presenting macrophages and dendritic cells. A vector armed with this gene causes the tumor to attract and activate immune cells, leading to an anti-tumor immune response. GM-CSF’s role in enhancing OVs is well documented, with most authors concluding that the molecule helps to re-educate tumor-associated macrophages from wound healing M2 subtypes to pro-inflammatory and immune stimulating M1 forms [50]. GM-CSF is expressed by HSV1 vector TVEC/IMLYGIC, Vaccinia vectors VG9-GMCSF-IL24, and PexaVec, as well as NDV MEDI5395 and others [51,52,53]. A commonality between antibody and cytokine-armed vectors is the recruitment of T cells to sites of vector infection. The choice to use either or both is an active pursuit of many labs, and these two groups are in no way mutually exclusive. 

Another arming gene that recruits myeloid lineages is the fms-related tyrosine kinase 3 ligand (FLT3L). FLT3L is particularly useful for developing plasmacytoid dendritic cells, as well as the differentiation of hematopoietic cells in bone marrow [54]. The presence of plasmacytoid dendritic cells promotes a pro-inflammatory milieu which activates effector immune populations leading to viremic control [55], which could translate to tumor response via a bystander effect. G47Δ-Flt3L, OCR177, superior killing virus (SKV), and E3LΔ83N encode FLT3L and cause robust T-cell infiltration to tumors in mouse models and clinic practice; however, their combination with other transgenes such as anti-CTLA4 and IL-12 belies that FLT3L expression by itself is not always potent enough to enhance oncolytic activity [56,57]. 

Although not the focus of many immunotherapy conferences, NK cells can play a role in tumor clearance. UL16 binding protein 3 (ULBP3) is a ligand for the NK NKG2D receptor, which activates NK cells to kill and is sequestered to cloak HCMV from NK cell destruction [58,59]. For this reason, it was used as an arming gene in oHSVULBP3 for use in GBM therapy, along with IL-12. The vector recruits T cells, whereas mouse NK cells lack the receptor to bind human ULBP3, suggesting that a greater NK response could occur in patients, which may soon be studied [60].

One innate inflammatory cytokine that performs well on its own is interferon-β (IFNβ). Interferons are immune stimulatory genes which actively hamper viral infection by halting protein synthesis, inhibiting cell growth, and activating nearby immune cells. Interestingly, many of the OV platforms used today were inadvertently developed to be extremely sensitive to interferons because they were developed in cancer cell lines that had lost IFN signaling or sensing mechanisms through culturing. Especially sensitive to IFN are RNA viruses, because their genomes often raise TLR-mediated detection and activation of the IFN pathway [61]. Paradoxically, arming oncolytic VSV, an ssRNA virus, with IFNβ results in similar viral replication within tumors, but increases and maintains T-cell-mediated tumor reduction. Interestingly, in animal melanoma models, the treated subjects maintained more active T cells in combination with anti-PD1 treatment, which the authors claim was due to the transgene’s reversal of T-cell exhaustion [62]. Currently, several clinical studies are under way using systemic injections of VSV-IFNβ to test its efficacy, based originally on myeloma research [20]. Administration of an OX40 agonist led to similar mouse survival in these VSV experiments, once more denoting the central reliance on effector T cells for tumor clearance. The inclusion of IFN within the vector may cause the upregulation of innate immune signaling which is absent in many tumor cells [63]. Restoration of IFN to cancer cells would then combat the immunosuppressive nature of the tumor microenvironment by upregulating cell stress signals which serve to activate immune cells, making these vectors potentially potent deprogrammers of the tumor microenvironment. Furthermore, the secretion of interferons from virally infected cells would have a positive role in maintaining T cell responses against the tumor [63]. Of note for dog lovers, VSV-IFNβ has been used to clear canine cancers [64]. 

This segues into a major subgroup of arming genes which explicitly cause effector differentiation and the homing of immune cells to the local tumor. OVs expressing interleukins, cytokines, and other attractants have been developed alongside those expressing antibodies. Interleukin-2 and -12 (IL-2, IL-12) were early arming genes incorporated after the discovery of T cell checkpoint antibody effectiveness with OVs. IL-2 generally causes T cell proliferation, and is given in tandem with many T-cell-based clinical cell therapies to promote engraftment and proliferation. This includes the differentiation of naïve T cells into Th1 or Th2 effectors, but also TREG development. The only dampening effect IL-2 has on T cell expansion is that it antagonizes Th17 differentiation [65]. Similarly, IL-12 is an immune-stimulatory cytokine, expressed by activated macrophages, dendritic cells, microglia, monocytes, neutrophils, and B cells. IL-12 signaling initiates the expression of interferon gamma (IFNγ), which is the primary activator of cytotoxic effector T cells [66]. Similar to the systemic use of checkpoint inhibitors and BiTEs, IL-2 and IL-12 use in humans has been limited by toxicity [67,68], and for IL-2’s expansion of suppressive TREG cells. IL-12 was first delivered using an adenovirus vector in 1999, after IL-12 was shown to potently cause tumor reduction, although it was also associated with toxicity when delivered systemically [69,70]. 

Several herpes vectors have added IL-2 or IL-12 expression cassettes which have increased survival and immune recruitment in murine models such as M002, M032, G47∆-IL-12, ONCR177, and NV1042. Aside from ONCR177, these vectors only express their one arming gene which results in an increase in efficacy, demonstrating IL-2 and IL-12’s ability to enhance potency, reviewed in [6,71,72,73]. Of note, R113 and R123 are wildtype HSV1 vectors that have been retargeted to HER2-expressing cells [9]. R123 expresses IL-12 and GM-CSF which, when injected into murine flank lung tumors, cause a much better response than the unarmed vector, and are curative with the addition of an anti-PD1 antibody. This efficacy is dependent on CD4+ and CD8+ T cells while being NK-cell-independent, demonstrating the boost that these arming factors give to tumor-infiltrating T cells, allowing them to overcome the immunosuppressive tumor environment and clear tumors. Of interest is how this vector is efficacious and safe despite retaining ICP34.5, relying solely on replacement of the natural HSV glycoproteins to enter tumor cells. 

The VACVs which incorporate the IL-2 or IL-12 genes do so in addition to other arming factors, making it difficult to ascertain the individual role of the interleukins. Cont-VV, a VACV derived from the LC16mO strain with VGF and O1L deletions and modification of B5R for reduced antigenicity, has an IL-12 and IL-17 expression cassette. Cont-VV-injected CT26 and LLC mouse tumors were reduced and even cleared in some cases in immunocompetent mice. In humanized NOG mice, both Cont-VV and its parental virus caused tumor clearance; however, only the armed-vector-injected tumor saw infiltration of CD4+, CD8+, and NK cells [74]. Similar to this dual strategy is vvDD-mIL2, which is given in addition to CpG treatment. Co-administration decreased MC38 murine colorectal flank tumor growth in a contralateral model. Immunological profiles in the spleen showed an increased CD8+ T cell/regulatory T cell ratio and increased CD11c+ cells after dual injection in one flank tumor [75]. As with most fields, IL-12’s early discovery as a potent anti-tumor agent has led to its incorporation into many vectors not discussed here, but which may interest the reader [40,76,77,78]. 

## 4. Vectors Expressing Transgenes Which Do Not Directly Interact with Immune Cells

Our final category covers some OVs which employ transgenes and tactics not entirely in support of the current dogma of oncolytic virology, but have achieved results nonetheless. Hyaluronidase breaks down hyaluronan, which is present in the tissue extra-cellular matrix (ECM). High amounts of hyaluronan are often correlated with metastasis and a poor prognosis in breast, pancreatic, and non-small cell lung adenocarcinomas [79]. This increased ECM density decreases immune cell infiltration and stymies cell-to-cell spread of the vector. To address this issue, several groups have armed their vectors with hyaluronidase [80,81,82,83]. VCN01 is a variant of the ICOVIR adenovirus (Ad-DM-E2F-K-Δ24RGD) with Ad-E1A under the E2F promoter control, instead of the myotonic dystrophy locus in the parental ICOVIR vector. VCN01 expresses the hyaluronidase-PH20 protein and contains a retargeting modification in its fiber [84]. One phase I trial has finished, using the vector (NCT02045589), while several others are ongoing. In the phase I trial, it was reported that after IT injection into pancreatic tumors with VCN-01, enhanced extravasation of Gemcitabine drugs was observed, suggesting ECM breakdown and an increase in the tumor’s permeability. PH20 expression and virus was detected in the blood up to 40 days after initial injection, as well denoting vector replication [85]. Several more phase I trials are exploring VCN-01’s safety and efficacy in head and neck squamous cell carcinoma and refractory retinoblastoma, highlighting that hyaluronidase-expressing vectors seek to spread widely rather than focus on a specific cancer tissue to target. 

Another combination which has potential for OV therapies is the use of BRAF and MEK inhibitors (BRAFi, MEKi) to enhance oncolytic activity. Both inhibitors were recently discovered to enhance oncolytic herpes simplex vector efficacy in tumor models. MEK inhibition limits the activation of ERK, and subsequently, the Ras/MAPK pathway. Inhibition leads to reduced cell proliferation but an increase in PKR expression, which dampens HSV1 replication [86]. In vitro, MEKi synergized with TVEC to kill human melanoma lines, but did not translate to a B16 mouse model, due to B16 cells not being permissive for TVEC replication. A D4M3A mutation enables replication in these mouse cells, allowing researchers to test TVEC + MEKi in a D4M3A BRAFV600E melanoma model. In this and an SKMEL xenograft, MEKi synergized with TVEC, leading to increased rates of survival in a combination therapy over monotherapies, including re-challenge protection. Furthermore, a triple therapy (TVEC + MEKi + anti-PD1) worked better, which is important because all three are available for use in the clinic today [87]. Again, using a BRAFV600E model, this time for thyroid cancer, RP1 oHSV was shown to replicate better in the presence of BRAFi, kill better in vitro, and led to better survival rates in animal models. The survival benefit was also enhanced using double and triple therapy combinations of TVEC, BRAFi, and anti-CTLA4 + PD1 antibodies [88]. Collectively, these studies highlight that the utilization of small-molecule inhibitors in tandem with OVs and checkpoint inhibitors is well within reason, and should be actively pursued in the clinic.

## 5. Conclusions

At this point in OV development, there are no real conclusions about which pathway or dogma has a clear advantage over the others. This review has focused on transgenes which recruit immune cells to perform the majority of the oncolysis, leaving the vector itself as the initiator to these therapies, not the effector. Even this ideology is splintered, as old, unarmed vectors continue their use in clinical trials, some with surprisingly good results given their age and lack of previous efficacy [89,90]. To date, it has been difficult to objectively compare which arming genes are better due to differences in the viral platform and lack of direct comparisons. There are most likely certain tumors which are better suited for oHSVs rather than oAds or VACVs, and direct competition experiments could illuminate this and lead to research into what differences between the vectors and tumor biology cause this difference. This may ultimately lead to better vectors or more chimeric OVs, which should be encouraged. As more and more space is opened up for transgene insertion in OVs, so too is more of the original vector’s inherent replicative biology, providing options for greater and hopefully more potent combinations of transgenes. As the past decade has demonstrated, the oncolytic virology field is expanding upon its early successes, and will hopefully continue to expand in the fight to cure cancer.

## Figures and Tables

**Table 1:** Oncolytic Viruses in Clinical Trial. CSCC (cutaneous squamous cell carcinoma), TN breast cancer (triple-negative), CRC (colorectal cancer), NSCC (non-small cell carcinoma), NIS (sodium iodide symporter), rluc (renilla luciferase), β-gal (beta-galactosidase), β-gluc (beta-glucocordinase), FCU1 (fusion protein of cytosine deaminase and uracilphosphoribosyltransferase).

Vector	Clinical Trial	Phase	Oncolytic Virus	Transgene	Intervention	Tumor	Year
Herpes Simplex Virus-1	NCT03152318	1	rQNestin34.5v.2	-	Cyclophosphamide	Recurrent or progressive brain	2018
	NCT03747744	1	Talimogene Laherparepvec	GM-CSF	CD1c (BDCA-1) + MyDC	Melanoma	2018
	NCT03657576	1	C134	CMV-IRS1	-	Recurrent glioma/anaplastic astrocytoma/ gliosarcoma	2018
	NCT04050436	2	RP1	GM-CSF	Cemiplimab	Advanced and metastatic CSCC	2019
	NCT03911388	1	G207	-	5Gy radiation	Recurrent or refractory brain	2019
	NCT04185311	1	Talimogene Laherparepvec	GM-CSF	Ipilimumab and Nivolumab	TN breast cancer	2019
	NCT04348916	1	ONCR-177	IL-12, CCL4, FLT3LG, αPD-1, αCTLA-4	Pembrolizumab	Advanced and/or refractory solid tumors or liver metastases	2021
	NCT04427306	2	Talimogene Laherparepvec	GM-CSF	-	Melanoma prior to definitive excision	2020
	NCT04349436	1B	RP1	GM-CSF	-	SCCC	2020
	NCT04735978	1	RP3	GM-CSF, αCTLA-4, 4-1BBL, CD40L	Mono-clonal PD1 antibody	Advanced solid tumors	2021
Herpes Simplex-Virus-2	NCT03866525	1/2	OH2	GM-CSF	HX008	Solid tumors and gastrointestinal cancers	2019
	NCT04637698	1b/2	OH2	GM-CSF	-	Pancreatic cancer	2020
	NCT04386967	1/2	OH2	GM-CSF	Alone and with Keytruda	Advanced melanoma	2020
	NCT04616443	1B/2	OH2	GM-CSF	HX008	Advanced melanoma	2020
Adenovirus	NCT03004183	2	ADV/HSV-tk	HSV1-TK	Valacyclovir, 30 Gy radiation, Pembrolizumab	Metastatic NSCC and TN breast cancer	2016
	NCT02705196	1/2	LOAd703	TMZ-CD40L, 4-1BBL	Gemcitabine, nab-paclitaxel	Pancreatic cancer	2016
	NCT03003676	1	ONCOS-102	GM-CSF	Cyclophosphamide and Pembrolizumab	Advanced or unresectable melanoma	2016
	NCT03225989	1/2	LOAd703	TMZ-CD40L and 41BBL	Standard of care chemotherapy *	Pancreatic, biliary, colorectal and ovarian cancers	2017
	NCT03714334	1	DNX-2440	-	-	Recurrent glioblastoma	2018
	NCT03740256	1	CAdVEC	-	-	HER2-positive cancers	2018
	NCT03896568	1	DNX-2401	-	-	Recurrent glioblastoma	2019
	NCT03178032	1	DNX-2401	-	-	DIPG and cerebellar tumors	2019
	NCT03852511	1	NG-350A	α-CD40	-	Advanced or metastatic epithelial tumors	2019
	NCT03916510	1	Enadenotucirev	-	Capecitabine and 50 Gy radiation	Advanced rectal cancer	2019
	NCT04053283	1	NG-641	FAP TAc antibody	-	Metastatic or advanced epithelial tumors	2019
	NCT04097002	1/2	ORCA-010	-	-	Prostate adenocarcinoma	2019
	NCT04391049	1	OBP-301—Telomerase-specific Type 5	-	Carboplatin and Paclitaxel and radiation	Esophageal and gastroesophageal cancer with local invasion or regional structures invasion	2020
	NCT04217473	1	(TILT-123)	TNF-alpha, IL-2	Tumor-infiltrating lymphocytes	Metastatic melanoma	2020
	NCT04673942	1	AdAPT-001	-	-	Malignant solid tumors	2020
Measles Virus	NCT02068794	1/2	MV-NIS	NIS	-	Ovarian, primary peritoneal or fallopian tube	2014
	NCT02364713	2	MV-NIS	NIS	-	Ovarian, fallopian, or peritoneal cancer	2015
	NCT03171493	1	MV-NIS	NIS	-	Urothelial carcinoma	2017
Vaccinia Vector	NCT02977156	1	Pexa–Vec	GM-CSF, β-gal	Ipilimumab	Metastatic tumor	2016
	NCT02759588	1/2	GL-ONC1	rLuc, β-gal, β-gluc	Bevacizumab	Recurrent/refractory ovarian cancer and peritoneal carcinomatosis	2016
	NCT03294486	1/2	TG6002	FCU1	5-Flucyrosine	Glioblastoma	2017
	NCT03206073	1/2	Pexa-Vec	GM-CSF, β-gal	Tremelimumab and Durvalumab	Metastatic colorectal cancer	2017
	NCT03294083	1/2	Pexa-Vec	GM-CSF, β-gal	Cemiplimab	Renal cell carcinoma	2017
	NCT03954067	1	ASP9801	IL-7, IL-12	-	Solid metastatic cancers	2019
Reovirus	NCT03605719	1	Pelareorep	-	Dexamethasone, carfilzomib, and nivolumab	Recurrent multiple myeloma	2018
	NCT04445844	2	Pelareorep	-	Retifanlimab	Metastatic triple-negative breast cancer	2018
	NCT04102618	1	Pelareorep	-	Trastuzumab, Letrozole Atezolizumab	Breast cancer	2019
Polio Virus	NCT03043391	1	PVSRIPO	-	-	Recurrent WHO Grade III and IV gliomas (2–12 yo)	2017
Maraba Virus	NCT03773744	1	Ad/MG1-MAGEA3	-	Pembrolizumab	Metastatic melanoma and squamous skin cell carcinoma	2018
Newcastle Disease Virus	NCT03889275	1	MEDI5395	GM-CSF	Durvalumab	Advanced solid tumors	2019

* Standard of care based on the primary investigator.
